# 88462 Fluconazole distribution in CNS and gynecological tissues in HIV-related cryptococcal meningitis decedents

**DOI:** 10.1017/cts.2021.648

**Published:** 2021-03-31

**Authors:** Melanie R. Nicol, Fan Wang, Ambayo Richard, Olivie Carolyne Namuju, Katelyn A Pastick, David R Boulware, David B Meya, Robert Lukande

**Affiliations:** 1 University of Minnesota College of Pharmacy; 2 Makerere University Infectious Disease Institute; 3 University of Minnesota Medical School; 4 Makerere University

## Abstract

ABSTRACT IMPACT: Plasma and CSF are not reliable estimates of drug exposure in tissue compartments relevant for treatment and prevention of infectious diseases. OBJECTIVES/GOALS: Globally, high dose fluconazole is widely used in the management of cryptococcal meningitis. While it is known to readily penetrate into cerebrospinal (CSF), less is known about drug concentrations in brain parenchymal tissues. Similarly, distribution of fluconazole into gynecological tissues has not been robustly characterized. METHODS/STUDY POPULATION: With informed consent from next-of-kin, we conducted autopsies within 24h of death for hospitalized Ugandans receiving fluconazole for treatment or secondary prophylaxis of cryptococcal meningitis. Dosing history was abstracted from medical chart and caregiver interviews. Fluconazole concentrations were determined using high-performance liquid chromatography- tandem mass spectrometry (LC-MS/MS) in plasma, CSF, 10 brain compartments (frontal, parietal, and occipital lobes, corpus callosum, globus pallidus, hippocampus, midbrain, medulla oblongata, spinal cord, and choroid plexus) and 4 female genital compartments (cervix, vagina, ovary, and uterus), depending on tissue availability. Descriptive statistics of tissue to plasma ratios were used to describe concentrations relative to plasma. RESULTS/ANTICIPATED RESULTS: Fluconazole concentrations were measured in available tissues of 21 individuals with detectable fluconazole in plasma. Daily doses of fluconazole were 200 mg (n=4), 400 mg (n=1), 800 mg (n=4), 1200 mg (n=9) or unknown (n=3). CSF concentrations (n=10) ranged from 93-1380% (median 100%) of plasma while brain concentrations (n=3) across all 10 compartments ranged from 45% to 89% (median 69%) of plasma. In the female genital tract, cervical concentrations (n=10) were 9-78% (median 65%) of plasma and in the 2 individuals with available tissue, concentrations in vaginal, ovarian, and uterine tissues were similar to cervix, ranging from 63-105% of plasma. DISCUSSION/SIGNIFICANCE OF FINDINGS: Measuring drug concentrations directly in tissues, the presumed site of action, improves estimates of drug efficacy. While fluconazole concentrations in CSF were similar to plasma, actual brain tissues were consistently lower. Concentrations were similar between upper and lower female genital tracts, but were consistently lower than plasma.

